# The Effect of Drug Selection on Pediatric Rapid Sequence Induction Success: A Systematic Review and Meta-Analysis

**DOI:** 10.7759/cureus.87016

**Published:** 2025-06-30

**Authors:** Ali Abdelaal, Zubair Ahmed

**Affiliations:** 1 Anesthesia, Walsall Manor Hospital, Walsall, GBR; 2 Neuroscience, University of Birmingham, Birmingham, GBR

**Keywords:** etomidate, pediatrics, propofol, rapid sequence intubation (rsi), rocuronium

## Abstract

The purpose of this systematic review is to evaluate the effect of drug selection on endotracheal intubation conditions, hemodynamic response, and time course of neuromuscular blockade in children undergoing rapid sequence intubation (RSI). We systematically searched databases with no time restrictions for studies related to pediatric RSI and induction agents, as well as neuromuscular blockers. The inclusion criteria consisted of randomized controlled trials (RCTs) that studied pediatric patients who had undergone RSI. Studies involving any number of adult patients were excluded, as were studies that included opioids as induction agents. Seven RCTs (520 participants) were included. Five of the studies reported primary outcomes related to endotracheal intubation conditions, with four of these using identical methods for recording jaw relaxation, vocal cord positioning, and diaphragmatic response. Subgroup meta-analysis of these four studies reported an odds ratio of 3.38 (95% confidence interval, 1.45-7.90) for excellent intubating conditions, favoring rocuronium over other neuromuscular blockers. This meant that patients given rocuronium were 3.38 times more likely to have excellent intubating conditions compared to other neuromuscular blockers, and that this result was not due to chance. Suxamethonium consistently had faster onset and recovery times. Our systematic review demonstrated that rocuronium provides superior intubating conditions, allowing for improved first-pass success rates in pediatric RSI. Future studies should evaluate the effect of different induction agents on endotracheal intubation conditions and whether the RSI procedure itself improves mortality compared to other intubation methods.

## Introduction and background

Rapid sequence intubation (RSI) is a standardized protocol for intubating a critically ill patient using an intravenous anesthetic agent, a neuromuscular blocker, and an endotracheal tube to induce unconsciousness and laryngeal muscle paralysis, thereby securing the airway [1,2]. Most induction agents, such as propofol and etomidate, act mainly by binding to γ-aminobutyric acid receptors and exacerbate the homeostatic inhibition of neural systems; on the contrary, ketamine predominantly acts to antagonize N-methyl-D-aspartate receptors [3,4]. Neuromuscular blockers can be divided into depolarizing and nondepolarizing subgroups; the former acts as acetylcholine receptor agonists, leading to persistent activation that prevents repolarization (causing flaccid paralysis), and the latter acts as competitive acetylcholine receptor antagonists, leading to depolarization block ​[5,6].

RSI is indicated in patients with unknown or inadequate fasting status (e.g., in an unconscious or obtunded patient), as the inflatable cuff on the endotracheal tube prevents regurgitation of stomach contents and, therefore, mitigates aspiration ​[7,8]. Furthermore, RSI is indicated if the patient lacks intact swallow and gag reflexes and if the healthcare professional requires immediate protection of the patient’s airway. The idea of an “ideal” anesthetic agent for use in RSI has surfaced time and time again to no avail; therefore, the logical next step is to use a combination of “preferred” drugs to induce unconsciousness and unresponsiveness, while being quickly metabolized or reversed with medication and having few side effects ​[4,9,10].

In adults, numerous systematic reviews exist that compare different modifications of RSI, including controlled vs. classic RSI, various induction agents such as etomidate vs. ketamine, and different neuromuscular blockers, such as suxamethonium (succinylcholine) vs. rocuronium ​[11-16]. These reviews present conflicting information due in some respects to a lack of patient numbers and the low quality of primary studies. However, no such systematic reviews have been published to date that focus on the pediatric population. Between 2018 and 2019, almost 4.7 million patients attended Accident and Emergency departments as emergency admissions in England alone [17]. Of these, the majority of returning emergency department visitors were made up of young children and elderly patients over the age of 80, with both demographic groups being significantly more likely to return to the emergency department than middle-aged patients ​[18,19]. Children make up a small yet significant percentage of total patients receiving RSI; however, the percentage of high-risk patients undergoing RSI is similar in both adults and children. One study suggests that patients presenting with acute abdominal complaints are intubated using RSI techniques in 88.6% of both adults and children [20,21]. The lack of systematic reviews on the ideal combination and dose of RSI drugs in children is a glaring omission in the literature, particularly as this is such a common and important emergency procedure, and one which this dissertation will address.

This systematic review aims to compare the efficacy, safety, and optimal dosing of different induction agents and neuromuscular blockers with regard to pediatric RSI to determine whether there is one drug that performs best over others in its class.

## Review

Methods

Protocol and Registration

This systematic review follows the Preferred Reporting Items for Systematic Reviews and Meta-analysis (PRISMA) guidelines but was not registered with the PROSPERO database ​[22].

Literature Search Strategy

The literature search for this systematic review was conducted in accordance with the PRISMA guidelines ​[22]. The author searched four databases, MEDLINE, Embase, Web of Science, and The Cochrane Library, using their respective websites with searches up to August 9, 2022, to include as many primary studies as possible. By using multiple databases, the occurrence of reporting bias was reduced. Although the inclusion of Google Scholar would have provided the widest coverage of studies, the combination of MEDLINE, Embase, and Web of Science alone covered 95.9% of all possible studies published, which was deemed sufficient ​[23]. The search was conducted on August 9, 2022. Medical Subject Headings (MeSH) terms were used with Boolean operators as follows: (Paediatric OR Children) AND (Rapid Sequence Intubation) AND (Induction OR Sedation).

A Population, Intervention, Comparator, and Outcome framework was used to synthesize a review question. The population included pediatric patients (defined as those under 18) who underwent RSI. The age of 18 was chosen to eliminate any confusion arising from countries defining the age of adulthood as 21. Intervention and comparators were defined as different induction agents or neuromuscular blockers, or different doses by weight. Outcomes for the studies would include endotracheal intubation conditions as well as time to intubation and time to recovery. Therefore, the review question posed was as follows: What is the most effective drug selection for pediatric RSI success?

To assess the effectiveness of different drug selections on pediatric RSI success, the most appropriate study design to undertake was a systematic review with a concurrent meta-analysis. This allowed for the identification of primary studies using rigorous frameworks, the assessment of the risk of bias in each study, the extraction and analysis of all data, and a discussion incorporating all the data together ​[24,25]. While a double-blind randomized controlled trial (RCT) would have been a good alternative, the ethical approval required and the duration of data collection would have been beyond the scope of time available to carry out this research. Furthermore, the integration of data from many alternative RCTs would not have been available.

Inclusion and Exclusion Criteria

Inclusion criteria for title and abstract screening at the time of literature search included all studies that measured the effectiveness of induction agents and neuromuscular blockers on pediatric RSI success, measured via any outcome. Exclusion criteria for studies included in the meta-analysis and risk of bias analyses were as follows: 1) studies where the method of intubation was not RSI; 2) studies involving any adult patients; 3) studies involving opioids used as the main induction agent; 4) studies not in English; 5) studies where the full text was inaccessible; and 6) studies that were not RCTs. Studies involving opioids were not included, as this could potentially confound results due to their effects on airway reflexes and hemodynamics.

The removal of a non-English study may have led to a language bias; however, this has been shown to have a minimal effect on overall conclusions in recent studies [26,27]. One study was not included due to inability to access the full text; the author of this systematic review’s supervisor (ZA) kindly reached out to the author of the study for access, but the study would have been removed at the end of the literature search regardless, as it was not an RCT. An explanation of the data collection process is written below.

Study Screening and Selection

From the studies found in the initial search, all reference lists were screened to identify any potential gray literature that could be related. All the studies found including the initial search and studies from reference lists were title and abstract screened by the author; there was a chance that not enough RCTs existed to conduct a meta-analysis, and therefore, this systematic review would have included data from the next best form of evidence, namely cohort and then case-control studies. The author’s supervisor kindly aided with the search to include or exclude some studies where the author was uncertain as to whether they should be included. However, seven RCTs were collated; therefore, the non-RCTs were removed to compile a meta-analysis using high-quality data with a low risk of bias. Primary outcomes extracted from the studies included endotracheal intubation conditions. Secondary outcomes extracted included hemodynamic response to laryngotracheal intubation (LTI), train-of-four (TOF) electrode measurements, and time course of neuromuscular blockade. An Excel spreadsheet (Microsoft Corporation, Redmond, WA) was used to tabulate the data used, with means and standard deviations included.

Risk of Bias

Risk of bias in the resultant seven RCTs was assessed using the Cochrane risk-of-bias tool for randomized trials (RoB2). Two authors (AA/ZA) independently conducted their assessment of each study, grading the following five domains as low, some concerns or high risk of bias: 1) risk of bias arising from the randomization process, 2) risk of bias due to deviations from the intended interventions, 3) risk of bias arising due to missing outcome data, 4) risk of bias arising in measurement of the outcome, and 5) risk of bias in selection of the reported result. These domains had structured guidelines to follow, which dictated how to grade the risk of bias. Each study was then graded for an overall risk of bias depending on the criteria mentioned in the guidelines.

Statistical Analysis

Assessment of heterogeneity was performed by examining the differences across studies for methodological heterogeneity. We used Review Manager (RevMan 5.3, Cochrane Informatics & Technology, London, UK) to determine the Q and I^2^ statistics (in percentages) to establish variation between the studies attributed to heterogeneity. A meta-analysis was conducted using RevMan 5.3 with the dichotomous data function, employing a random-effects model.

Results

Study Selection

**Figure 1:**
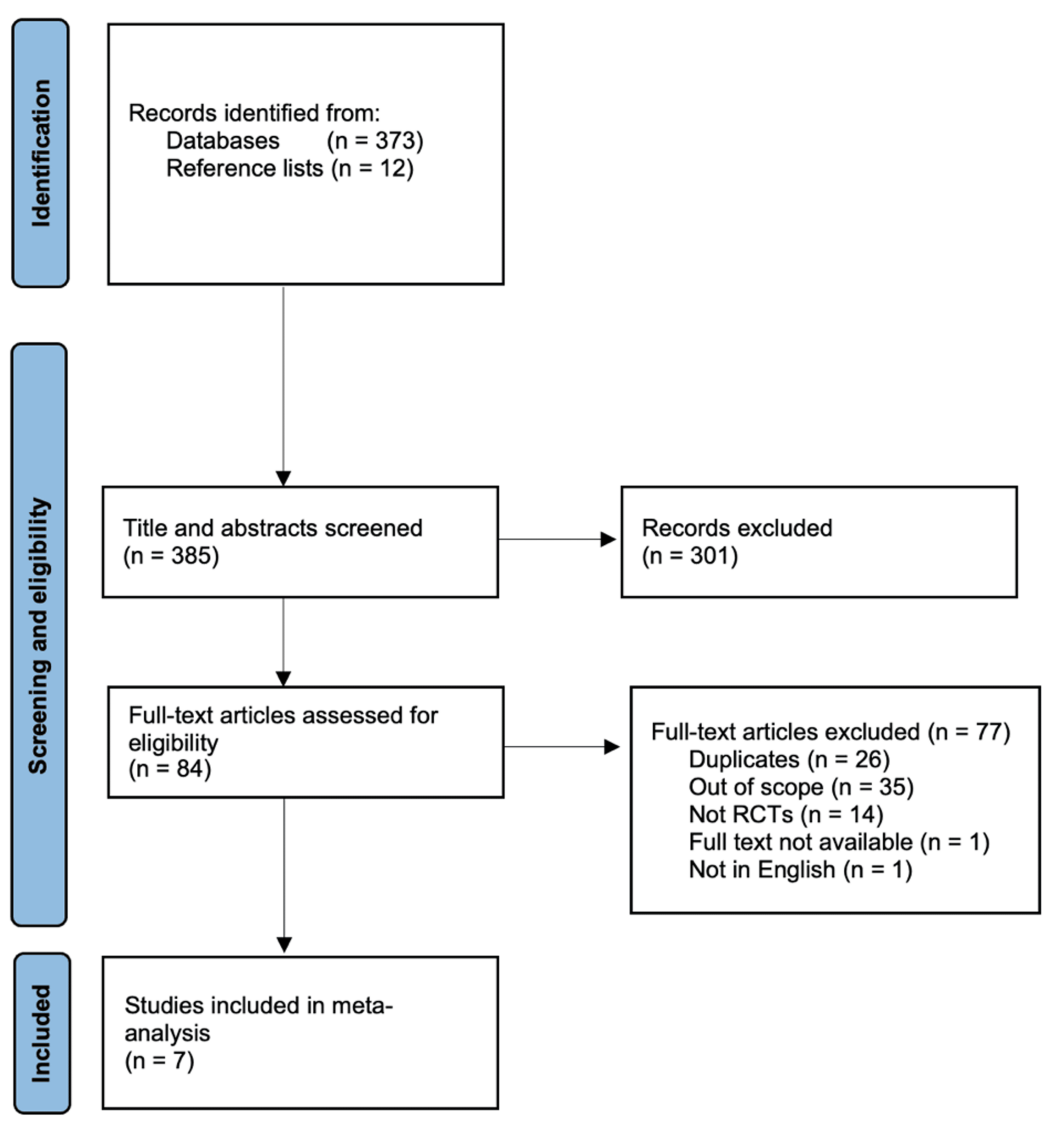
PRISMA flowchart for article selection PRISMA: Preferred Reporting Items for Systematic Reviews and Meta-analysis; RCT: randomized controlled trial Image credit: This is an original image created by the author Ali Abdelaal

During the initial search, 373 studies were identified using the MeSH terms above. A further 12 studies were found from the reference lists of these 373 studies. As stated previously, all studies were included in the title and abstract screening process to ensure that there were enough high-quality RCTs included. Three hundred one studies were excluded that fell into the aforementioned exclusion criteria, leaving 84 studies eligible for full-text screening. Of these, 77 articles were removed from the studies included in the meta-analysis, leaving seven RCTs. A PRISMA flowchart denoting the above process is shown in Figure 1.

Study Characteristics

Out of the seven RCTs included in this systematic review, none were published in the last decade [28-34]. A range of publishing dates from 1994 to 2010 was identified, with the median being 1998. The majority of the studies’ primary outcome measure was identified as endotracheal intubation conditions. Out of these five studies, four employed identical methods for assessing endotracheal intubation conditions, utilizing jaw relaxation, vocal cord positioning, and diaphragmatic response [28,30,31]. A range of countries were identified across the studies, the most notable being Hong Kong and Saudi Arabia, which each had the highest number of participants, at 120 pediatric patients each.

All studies compared the same induction agents or neuromuscular blockers at different doses, or different induction agents and neuromuscular blockers. Six of the studies used rocuronium either as an intervention or a comparator, with five of these studies using either a dose of 0.6 or 0.9 mg/kg. Eich et al. compared the effect of RSI-controlled vs. RSI-classic on the incidence of hypoxemia, intubation difficulties, and forced mask ventilation [33]. Although they used a pediatric simulator for induction, this was still included in the risk of bias assessment. However, no endotracheal intubation conditions were measured, so this study was excluded from the meta-analysis. Only one nonblinded and one single-blinded RCT were included, with the other five being double-blinded RCTs. In total, 490 real pediatric patients and 30 simulated pediatric patients were included, resulting in a total of 520 participants in the study. Table 1 shows the full study characteristics of the included studies.

**Table 1:** Characteristics of studies included in this systematic review RCT: randomized controlled trial; ASA: American Society of Anesthesiologists; RSI: rapid sequence intubation; LTI: laryngotracheal intubation

Study	Location	Study design	Participants	Intervention	Comparator
Lee et al. [28]	Korea	Double-blind RCT	65 children	Rocuronium (0.6 mg/kg) infusion, followed by propofol (2.5 mg/kg) infusion 20 seconds later	Propofol (2.5 mg/kg) infusion for 10-20 seconds, then rocuronium (0.6 mg/kg) infusion
Cheng et al. [29]	Hong Kong	Double-blind RCT	120 children	Rocuronium (0.6 mg/kg); rocuronium (0.9 mg/kg)	Suxamethonium (1.5 mg/kg)
Mazurek et al. [30]	USA	Double-blind RCT	26 children (with ASA grades I-III scheduled for emergency procedures)	Thiopental (5 mg/kg) and succinylcholine (1.5 mg/kg)	Thiopental (5 mg/kg) and Rocuronium (1.2 mg/kg)
Naguib et al. [31]	Saudi Arabia	Double-blind RCT	120 children (with ASA grade I)	Mivacurium, rocuronium, and mivacurium with rocuronium	Suxamethonium
Fuchs-Buder and Tassonyi [32]	UK	Double-blind RCT	100 children (ASA grades I-II)	Rocuronium (0.9 mg/kg)	Rocuronium (0.6 mg/kg)
Eich et al. [33]	Germany	Nonblinded RCT	30 adult trainees	RSI-controlled	RSI-classic
Schrum et al. [34]	USA	Single-blind RCT	59 children	LTI with propofol, LTI with halothane	LTI with thiopental

Appraisal of Studies

Intubating conditions: Five studies presented data on intubating conditions, with four studies demonstrating excellent intubating conditions. Mazurek et al. compared rocuronium 1.2 mg/kg to suxamethonium 1.5 mg/kg [30]. No significant differences were found between clinically acceptable intubating conditions (consisting of “excellent” and “good” intubating conditions) (p = 1.0) or only excellent intubating conditions (p = 0.41).

Two studies mentioned the incidence of unsafe RSI actions and hemodynamic response to LTI as their primary outcomes (Table 2) [33,34]. For example, Eich et al. compared RSI-classic vs. RSI-controlled, where “classic” included thiopentone, suxamethonium 2 mg/kg, cricoid pressure, and no mask ventilation, and “controlled” included thiopentone, rocuronium 0.6 mg/kg, no cricoid pressure, but with mask ventilation [33]. Hypoxemia occurred 100% of the time across the 15 simulated pediatric patients in the RSI-classic group; in contrast, this hypoxemia was not observed in the RSI-controlled group. No significant differences were found between the groups in the incidence of prolonged or unsuccessful intubation attempts, endobronchial intubations, or esophageal intubations. Schrum et al. discovered a lower hypertensive response to intubation in those who received propofol 3 mg/kg and halothane than in those who received either thiopental 5 mg/kg and halothane or halothane alone (p < 0.001) [34].

**Table 2:** Primary outcome measures of included studies RSI: rapid sequence intubation; LTI: laryngotracheal intubation

Study	Primary outcome measure
Lee et al. [28]	Intubating conditions
Cheng et al. [29]	Intubating conditions
Mazurek et al. [30]	Intubating conditions
Naguib et al. [31]	Intubating conditions
Fuchs-Buder and Tassonyi [32]	Intubating conditions
Eich et al. [33]	Incidence of unsafe RSI actions or signs
Schrum et al. [34]	Hemodynamic response to LTI

**Figure 2:**
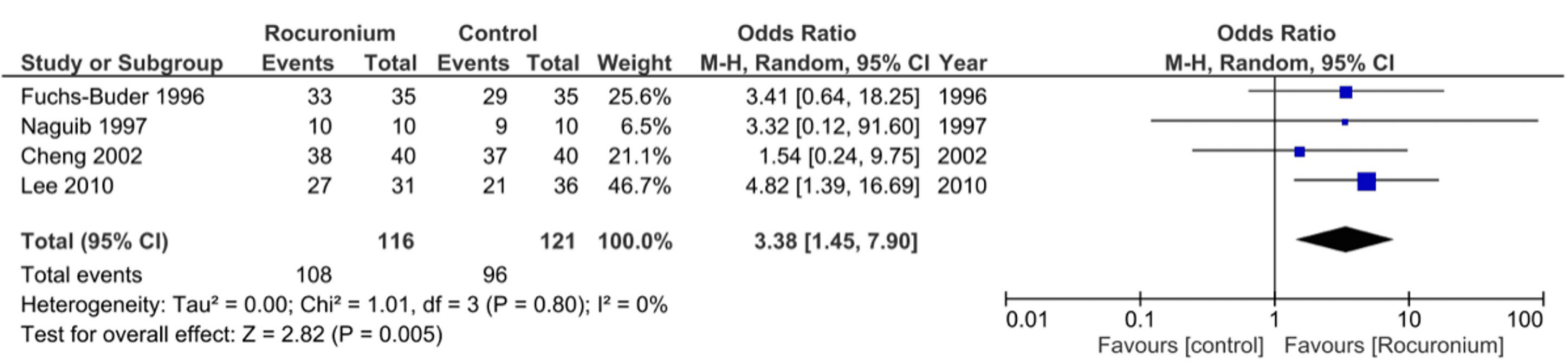
Meta-analysis for the number of excellent intubation events with rocuronium vs. other control measures in pediatric patients CI: confidence interval Image credit: This is an original image created by the author Zubair Ahmed

Subgroup analysis: A subgroup meta-analysis of four studies that reported excellent intubating conditions demonstrated an overall odds ratio of 3.38, 95% confidence interval (CI) of 1.45-7.90, I^2^ = 0%, and p = 0.005, in favor of rocuronium compared to other control drugs (Figure 2).

Other outcomes: A variety of other outcomes were also reported in these studies, including hemodynamic observations, time course of neuromuscular blockade, and TOF count (Table 3).

**Table 3:** Secondary outcome measures of included studies N/A: not available; paO_2_: partial pressure of oxygen; TOF: train of four; HR: heart rate; BP: blood pressure; EtCO_2_: end-tidal carbon dioxide

Study	Secondary outcome measures
Lee et al. [28]	N/A
Cheng et al. [29]	Identify paO_2_, HR, BP, EtCO_2_, and temperature; identification of RSI side effects
Mazurek et al. [30]	Disappearance of TOF electrode response; time to return of muscle twitch; 25% of TOF (return of TOF response to 25% of response before neuromuscular blockade)
Naguib et al. [31]	TOF count at time of intubation; time course of neuromuscular blockade by drug
Fuchs-Buder and Tassonyi [32]	Intubation time; time course of rocuronium-induced neuromuscular blockade
Eich et al. [33]	Providers’ physical or mental signs of stress (ergospirometry, salivary cortisol, and a-amylase, posttrial questionnaire)
Schrum et al. [34]	Emergence time from intubation; recovery times (time to Steward score of 6); discharge times; infant behavior following discharge

Cheng et al. studied hemodynamic variables as well as endotracheal intubation conditions [29]. The lowest range from baseline systolic arterial pressures, mean arterial pressures, and heart rate was found in the rocuronium 0.9 mg/kg group compared to the rocuronium 0.6 mg/kg and suxamethonium 1.5 mg/kg groups. However, there were no significant differences between the changes in these groups. One patient in the rocuronium 0.9 mg/kg group suffered from bronchospasm during intubation, which resolved during the RSI procedure with no direct treatment.

p values for time to intubation and time to onset of apnea in Mazurek et al.’s study were 0.5 and 0.8, respectively [30]. However, p values for time to twitch return and time to recovery of 25% TOF were both 0.0001; therefore, these are statistically significantly different between the groups.

Naguib et al. found that the time to onset of intubation was fastest in the suxamethonium 1 mg/kg group compared to all the other groups, although this was not statistically significant [31]. Duration of intubation and recovery index were also lowest in the suxamethonium 1 mg/kg group, again with no statistical significance.

Clinically acceptable intubating conditions (both excellent and good ratings) were statistically similar in both high and low doses of rocuronium (0.9 and 0.6 mg/kg) according to Fuchs-Buder and Tassonyi [32]. Statistically significant differences between the groups arose in onset time, muscle twitch height at one minute, clinical duration, and duration until recovery to 75% twitch height.

Secondary outcomes for Eich et al. are not related to this systematic review, so they are excluded (adult trainee physical or mental signs of stress) [33].

Schrum et al. delineated that propofol 3 mg/kg and halothane, and halothane alone, administered to infants aged one to six months showed a statistically significantly shorter extubating time than those given thiopental 5 mg/kg and halothane (p = 0.001) [34]. All other results were statistically similar. Some side effects were noted in all groups; however, no analysis was performed to show if these were statistically different or not. No differences in infant behavior, irritability, or incidence of vomiting were noted between the groups after discharge.

**Figure 3:**
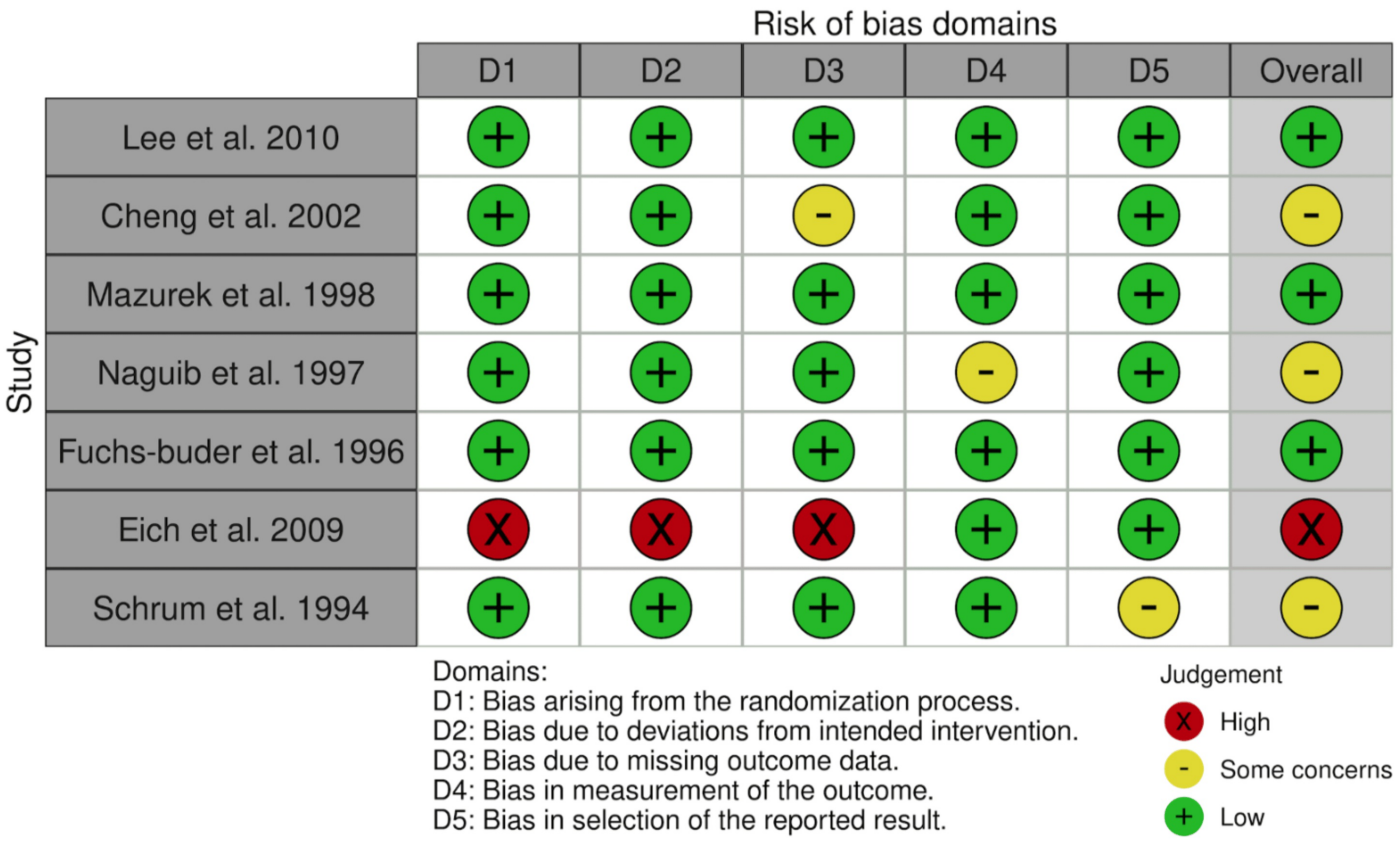
Traffic light plots visualizing the risk of bias assessment Image credit: This is an original image created by the author Ali Abdelaal

**Figure 4:**
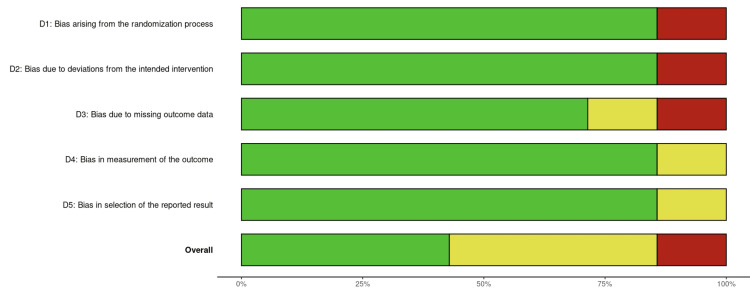
Weighted bar plots of distribution of risk of bias across domains Image credit: This is an original image created by the author Ali Abdelaal

Risk of bias assessment: Figures 3, 4 indicate the results of the risk of bias assessment using the RoB2 tool. Figure 3 depicts traffic light plots of domain-level judgments followed by an overall judgment of the risk of bias. There were no disagreements in the assessment of risk of bias between the author of the systematic review and their supervisor. The overall risk of bias for all included studies was deemed to be moderate. Some studies failed to blind personnel administering the intervention or control drugs, and one did not include the raw values of hemodynamic changes, preferring to plot unclear values on a graph.

Figure 4 shows weighted bar plots of the distribution of risk of bias assessments within the domains, followed by the overall risk of bias across included studies. Only 45% of the articles had a low risk of bias overall, with 17.5% of studies demonstrating a high risk of bias. In all the domains, at least 17.5% of studies had some concerns to high concerns, demonstrating a significant risk of bias in all the studies.

Discussion

Our systematic review shows that rocuronium 0.9 mg/kg is superior to other drugs in its class when it comes to endotracheal intubation conditions. This is the first systematic review and meta-analysis of its kind, with the aim of discerning which of the different RSI drugs used in a pediatric population provide the most successful outcomes.

Many systematic reviews and primary studies exist that pertain to different RSI drugs in adults. The “gold standard” neuromuscular blocker in the literature is suxamethonium, and rocuronium is usually compared to this ​[15,35]. In adults, suxamethonium provides superior conditions for intubation compared to rocuronium and has a faster recovery profile. Therefore, it should be used as the first line unless contraindications exist, such as in patients with hyperkalemia or those known to have low plasma cholinesterase activity. This is the case for traditional or modified RSI. Etomidate, ketamine, and propofol are all commonly used induction agents, yet studies have failed to conclusively show the superiority of one drug over the other ​[36]​; one systematic review has shown a benefit in using etomidate over ketamine to decrease postinduction hypotension ​[12]. A more recent retrospective multicenter trial showed no significant differences in hemodynamic effect between etomidate vs. ketamine vs. propofol ​[37].

However, children have altered physiology and behaviors with a predisposition to reflux of gastric contents and abdominal distension due to swallowing air compared to adults ​[7]. Anatomical considerations must also be taken into account in pediatric patients, such as a shorter and more anteriorly positioned airway. Conditions like macroglossia, cleft palate, and other craniofacial abnormalities can further complicate the process of RSI [38-41]. The first-pass success rate has been shown to be associated with smaller incidences of complications; therefore, creating optimal intubation conditions is paramount to improving the first-pass success rate and reducing complications associated with RSI ​[42,43].

For excellent intubating conditions, individual study point estimates of the treatment effect are on the same side of the line of no effect (i.e., are greater than one). There is moderate overlap of CIs, suggesting some heterogeneity, with most individual studies exhibiting a wide CI, indicating a less precise estimate of actual results [44]. However, pooling these data provides a much narrower CI, where the statistical power is increased ​[45]. This pooled data provide an overall effect size, which favors rocuronium over controls. Chi-square of 1.01 and degrees of freedom of 3 are associated with a p value of 0.80; therefore, differences between the observed and expected outcomes would occur by chance 80% of the time. Therefore, the hypothesis that rocuronium is superior to other neuromuscular blockers is accepted, and results favor rocuronium with the mean odds ratio (OR) = 3.38, 95% CI of 1.45-7.90, and p = 0.005. I^2^ = 0% suggests no heterogeneity between studies.

To deduce that rocuronium is the superior neuromuscular blocker over others in its class based on this systematic review alone is not without its consequences: only 520 pediatric cases were covered over the seven RCTs, resulting in 204 patients in the meta-analysis, which is by no means a large sample size. Rocuronium, while providing superior intubating conditions, also exhibits a longer recovery time than suxamethonium, which may be dangerous in a “can’t intubate, can’t ventilate” scenario, leading to hypoxemia and consequently hypoxia. However, with the introduction of sugammadex, which can rapidly reverse rocuronium-induced muscular blockade, the safety profile of rocuronium has improved massively [46]. Since children have a higher metabolic demand than adults, desaturation must be minimized. Preoxygenation with high-flow oxygen has been shown to offset this desaturation. Therefore, it should be actively enforced [47,48]. Rarely, allergies have been reported to be caused by rocuronium use. Otherwise, the drug is associated with minimal hemodynamic profile changes ​[49]. The dose of rocuronium must also be considered, as there are significant differences in intubating conditions with different doses administered.

Suxamethonium has been shown to cause asystole and bradycardia when used for RSI; atropine pretreatment is traditionally used to reduce the incidence of these complications. However, recently, studies have found no benefit in its use [50]. Furthermore, the risk of sudden cardiac death in seemingly “healthy” children with undiagnosed myopathies prevents doctors from using suxamethonium, preferring instead to use rocuronium ​[51].

The meta-analysis included in this systematic review only involved neuromuscular blockers; further studies, preferably RCTs, should be conducted on different induction agents and their respective optimal doses to determine if there is a single optimal agent that provides superior intubating conditions in children. If this single agent is identified, it could be balanced against clinical considerations to enable intubating professionals to achieve the best first-pass success rate and minimize complications. Additionally, the terms “modified” or “controlled” RSI are used differently by different healthcare professionals both nationally and internationally ​[52,53]. Attempting to define modified RSI universally will enable standardized research in the future, leading to safer practices and improved outcomes for patients requiring RSI.

One thing to note is that since RSI in an emergently unwell child is a very stressful procedure, both on the patient and the intubating professional, methods of reducing this stress should be examined. Whether RSI itself has improved mortality compared to other forms of intubation remains to be ascertained, particularly as adult patients selected for RSI may have less severe injuries and a better prognosis than those not selected for RSI ​[54]. Furthermore, the location where RSI is performed provides different outcomes, with emergency department RSI consistently producing a lower mortality rate than RSI performed in the prehospital setting [55]. To improve outcomes of pediatric patients, more studies should be conducted in children to determine whether there are differences in mortality and other measures between RSI and non-RSI intubation, and if the location of the RSI procedure impacts its success. For the latter, ideas such as prepackaging all the necessary drugs and equipment into a ready “RSI kit” for paramedics, such as those used in resuscitation trolleys, may improve outcomes in hostile environments or where there are diminished numbers of personnel. Educational interventions may be used to address this.

Limitations and Future Studies

All the studies in this systematic review and subsequent meta-analysis consisted of RCTs, which are at the top of the hierarchy of evidence triangle; however, none of them were published in the last decade. These studies have further limitations, which put into question their reliability. RSI was conducted by personnel of varying experience (mainly anesthetists) who may have inferior intubating skills in children compared to pediatric anesthetists, leading to altered endotracheal intubation conditions and subsequently outcomes. All the studies conducted, except for the one by Lee et al., measured the effects of different RSI drugs used on elective patients [28]. These patients may have altered baseline characteristics compared to those undergoing emergency procedures; therefore, this needs to be researched further. The technique and speed of intravenous injection may alter the conditions for endotracheal intubation, and there was no standardization across studies regarding the time points at which the drugs were injected ​[56].

Bias may have been introduced in the four studies included in the meta-analysis, where a doctor subjectively assesses endotracheal intubation conditions and rates them on a numerical scale, as shown in Figure 2. However, this should have been minimized with clinical experience and the fact that the assessor was blinded to the administered drug. An alternative method of measuring intubating conditions could potentially be utilized, using video laryngoscopy to assess vocal cord positioning or TOF electrodes to measure jaw relaxation.

There is a paucity of evidence in the pediatric population regarding RSI, which needs to be addressed; further studies involving high-quality RCTs should aim to standardize the timing of drug administration, delineate both the ideal method of intubation (whether RSI or otherwise) and a potential ideal induction agent, as well as improving outcomes of patients who have been intubated using RSI in nonemergency department settings. Studies including pediatric simulations, as shown by Eich et al., will also help deduce the optimal combination of induction agents and neuromuscular blockers. These simulations should be considered in study protocols [33].

## Conclusions

The study of RSI drug selection in pediatric patients has been neglected in recent years. This study contributes to the literature by suggesting that rocuronium 0.9 mg/kg be used as the first-line treatment in children who have no previous reactions to it. Barriers to implementation of this suggestion include existing hospital protocols where one induction drug or neuromuscular blocker is indicated in preference to another. Importantly, the intubating professional must consider the patient’s history, presenting complaints, allergies and likely prognosis to treat the patient holistically, using the available literature and their clinical experience on a case-by-case basis to serve the patient in their best interests.

## References

[1] Sinclair RF, Luxton MC (2005). Rapid sequence induction. Conti Educ Anaesth Crit Care Pain.

[2] Avery P, Morton S, Raitt J, Lossius HM, Lockey D (2021). Rapid sequence induction: where did the consensus go?. Scand J Trauma Resusc Emerg Med.

[3] Agarwala A, Dershwitz M (2011). Intravenous induction agents. Essential Clinical Anesthesia.

[4] Khan KS, Hayes I, Buggy DJ (2014). Pharmacology of anaesthetic agents I: intravenous anaesthetic agents. Cont Educ Anaesth Crit Care Pain.

[5] Jonas AA, Hunter JM (2004). Pharmacology of neuromuscular blocking drugs. Cont Educ Anaesth Crit Care Pain.

[6] Cook D, Simons DJ (2022). Neuromuscular Blockade. https://www.ncbi.nlm.nih.gov/books/NBK538301/.

[7] Newton R, Hack H (2016). Place of rapid sequence induction in paediatric anaesthesia. BJA Educ.

[8] Roshan R, Dhanapal SG, Joshua V, Madhiyazhagan M, Amirtharaj J, Priya G, Abhilash KP (2021). Aspiration during rapid sequence induction: prevalence and risk factors. Indian J Crit Care Med.

[9] Kaddoum R, Tarraf S, Shebbo FM (2022). Reduction of nonoperative time using the induction room, parallel processing, and sugammadex: a randomized clinical trial. Anesth Analg.

[10] Yu Y, Wang H, Bao Q, Zhang T, Chen B, Ding J (2022). Sugammadex versus neostigmine for neuromuscular block reversal and postoperative pulmonary complications in patients undergoing resection of lung cancer. J Cardiothorac Vasc Anesth.

[11] Yeung JK, Zed PJ (2002). A review of etomidate for rapid sequence intubation in the emergency department. CJEM.

[12] Sharda SC, Bhatia MS (2022). Etomidate compared to ketamine for induction during rapid sequence intubation: a systematic review and meta-analysis. Indian J Crit Care Med.

[13] de Carvalho CC, da Silva DM, de Athayde Regueira SL, de Souza AB, Rego CO, Ramos IB, Dos Santos Neto JM (2021). Comparison between rocuronium and succinylcholine for rapid sequence induction: a systematic review and network meta-analysis of randomized clinical trials. J Clin Anesth.

[14] Putzu A, Tramèr MR, Giffa M, Czarnetzki C (2020). The optimal dose of succinylcholine for rapid sequence induction: a systematic review and meta-analysis of randomized trials. BMC Anesthesiol.

[15] Tran DT, Newton EK, Mount VA, Lee JS, Mansour C, Wells GA, Perry JJ (2017). Rocuronium vs. succinylcholine for rapid sequence intubation: a Cochrane systematic review. Anaesthesia.

[16] Tessarolo E, Alkhouri H, Lelos N, Sarrami P, McCarthy S (2022). Review article: effectiveness and risks of cricoid pressure during rapid sequence induction for endotracheal intubation in the emergency department: a systematic review. Emerg Med Australas.

[17] (2022). A&E attendances and emergency admissions. https://www.england.nhs.uk/statistics/statistical-work-areas/ae-waiting-times-and-activity/.

[18] Greenfield G, Blair M, Aylin PP, Saxena S, Majeed A, Hoffman M, Bottle A (2020). Frequent attendances at emergency departments in England. Emerg Med J.

[19] Blair M, Poots AJ, Lim V (2018). Preschool children who are frequent attenders in emergency departments: an observational study of associated demographics and clinical characteristics. Arch Dis Child.

[20] Klucka J, Kosinova M, Zacharowski K (2020). Rapid sequence induction: an international survey. Eur J Anaesthesiol.

[21] Stevenson AG, Graham CA, Hall R, Korsah P, McGuffie AC (2007). Tracheal intubation in the emergency department: the Scottish district hospital perspective. Emerg Med J.

[22] Page MJ, McKenzie JE, Bossuyt PM (2021). The PRISMA 2020 statement: an updated guideline for reporting systematic reviews. BMJ.

[23] Bramer WM, Rethlefsen ML, Kleijnen J, Franco OH (2017). Optimal database combinations for literature searches in systematic reviews: a prospective exploratory study. Syst Rev.

[24] Gopalakrishnan S, Ganeshkumar P (2013). Systematic reviews and meta-analysis: understanding the best evidence in primary healthcare. J Family Med Prim Care.

[25] Loveman E, Jones J, Clegg AJ (2014). The clinical effectiveness and cost-effectiveness of ablative therapies in the management of liver metastases: systematic review and economic evaluation. Health Technol Assess.

[26] Morrison A, Polisena J, Husereau D (2012). The effect of English-language restriction on systematic review-based meta-analyses: a systematic review of empirical studies. Int J Technol Assess Health Care.

[27] Nussbaumer-Streit B, Klerings I, Dobrescu AI (2020). Excluding non-English publications from evidence-syntheses did not change conclusions: a meta-epidemiological study. J Clin Epidemiol.

[28] Lee SK, Hong JH, Kim AR (2010). Is the rapid sequence induction possible with 0.6 mg/kg rocuronium in pediatric patient?. Korean J Anesthesiol.

[29] Cheng CA, Aun CS, Gin T (2002). Comparison of rocuronium and suxamethonium for rapid tracheal intubation in children. Paediatr Anaesth.

[30] Mazurek AJ, Rae B, Hann S, Kim JI, Castro B, Coté CJ (1998). Rocuronium versus succinylcholine: are they equally effective during rapid-sequence induction of anesthesia?. Anesth Analg.

[31] Naguib M, Samarkandi AH, Ammar A, Turkistani A (1997). Comparison of suxamethonium and different combinations of rocuronium and mivacurium for rapid tracheal intubation in children. Br J Anaesth.

[32] Fuchs-Buder T, Tassonyi E (1996). Intubating conditions and time course of rocuronium-induced neuromuscular block in children. Br J Anaesth.

[33] Eich C, Timmermann A, Russo SG (2009). A controlled rapid-sequence induction technique for infants may reduce unsafe actions and stress. Acta Anaesthesiol Scand.

[34] Schrum SF, Hannallah RS, Verghese PM, Welborn LG, Norden JM, Ruttiman U (1994). Comparison of propofol and thiopental for rapid anesthesia induction in infants. Anesth Analg.

[35] Tran DT, Newton EK, Mount VA, Lee JS, Wells GA, Perry JJ (2015). Rocuronium versus succinylcholine for rapid sequence induction intubation. Cochrane Database Syst Rev.

[36] Baekgaard JS, Eskesen TG, Sillesen M, Rasmussen LS, Steinmetz J (2019). Ketamine as a rapid sequence induction agent in the trauma population: a systematic review. Anesth Analg.

[37] Leede E, Kempema J, Wilson C (2021). A multicenter investigation of the hemodynamic effects of induction agents for trauma rapid sequence intubation. J Trauma Acute Care Surg.

[38] Belanger J, Kossick M (2015). Methods of identifying and managing the difficult airway in the pediatric population. AANA J.

[39] Bachur R, Shaw K, Chamberlain J, Lavelle J, Nagler J, Shook JE (2016). Textbook of Pediatric Emergency Medicine.

[40] Karsli C, Pehora C, Al-Izzi A, Mathew P (2016). A retrospective review of pediatric difficult airways: once easy, not always easy. Can J Anaesth.

[41] Bai W, Golmirzaie K, Burke C, Van Veen T, Christensen R, Voepel-Lewis T, Malviya S (2016). Evaluation of emergency pediatric tracheal intubation by pediatric anesthesiologists on inpatient units and the emergency department. Paediatr Anaesth.

[42] Sakles JC, Chiu S, Mosier J, Walker C, Stolz U (2013). The importance of first pass success when performing orotracheal intubation in the emergency department. Acad Emerg Med.

[43] Hasegawa K, Shigemitsu K, Hagiwara Y, Chiba T, Watase H, Brown CA 3rd, Brown DF (2012). Association between repeated intubation attempts and adverse events in emergency departments: an analysis of a multicenter prospective observational study. Ann Emerg Med.

[44] Israel H, Richter RR (2011). A guide to understanding meta-analysis. J Orthop Sports Phys Ther.

[45] Cohn LD, Becker BJ (2003). How meta-analysis increases statistical power. Psychol Methods.

[46] Yang LP, Keam SJ (2009). Sugammadex: a review of its use in anaesthetic practice. Drugs.

[47] Pourmand A, Robinson C, Dorwart K, O'Connell F (2017). Pre-oxygenation: implications in emergency airway management. Am J Emerg Med.

[48] Hayes-Bradley C, Lewis A, Burns B, Miller M (2016). Efficacy of nasal cannula oxygen as a preoxygenation adjunct in emergency airway management. Ann Emerg Med.

[49] Mendez DR, Goto CS, Abramo TJ, Wiebe RA (2001). Safety and efficacy of rocuronium for controlled intubation with paralytics in the pediatric emergency department. Pediatr Emerg Care.

[50] Fleming B, McCollough M, Henderson HO (2005). Myth: atropine should be administered before succinylcholine for neonatal and pediatric intubation. CJEM.

[51] Tarquinio KM, Howell JD, Montgomery V (2015). Current medication practice and tracheal intubation safety outcomes from a prospective multicenter observational cohort study. Pediatr Crit Care Med.

[52] Ehrenfeld JM, Cassedy EA, Forbes VE, Mercaldo ND, Sandberg WS (2012). Modified rapid sequence induction and intubation: a survey of United States current practice. Anesth Analg.

[53] Abdallah C, Hannallah R (2014). Use of modified rapid sequence tracheal intubation in pediatric patients. Saudi J Anaesth.

[54] Cudnik MT, Newgard CD, Daya M, Jui J (2010). The impact of rapid sequence intubation on trauma patient mortality in attempted prehospital intubation. J Emerg Med.

[55] Fevang E, Perkins Z, Lockey D, Jeppesen E, Lossius HM (2017). A systematic review and meta-analysis comparing mortality in pre-hospital tracheal intubation to emergency department intubation in trauma patients. Crit Care.

[56] Cooper R, Mirakhur RK, Clarke RS, Boules Z (1992). Comparison of intubating conditions after administration of Org 9246 (rocuronium) and suxamethonium. Br J Anaesth.

